# Genome of *Bacillus cereus* strain *riobravensis*, a novel strain isolated from white sorghum dough made for human consumption

**DOI:** 10.1128/mra.00315-25

**Published:** 2026-03-24

**Authors:** Luis Ignacio Hernández-González, Erika Acosta-Cruz, Dafne Rodríguez-Medina, Rosa Issel Acosta-González, María Concepción Tamayo-Ordoñez, María Cristina Hernández-Jiménez, Guadalupe C. Rodríguez-Castillejos, Humberto Martínez-Montoya

**Affiliations:** 1 Unidad Académica Multidisciplinaria Reynosa Aztlán, Universidad Autónoma de Tamaulipas 27775, Reynosa, Tamaulipas, Mexico; 2 Departamento de Biotecnología, Facultad de Ciencias Químicas, Universidad Autónoma de Coahuila 27765, Saltillo, Coahuila, Mexico; University of Strathclyde, Glasgow, United Kingdom

**Keywords:** *Bacillus cereus*, genome, fermentation, probiotics

## Abstract

We report the draft genome sequence of *Bacillus cereus* strain *riobravensis*, isolated from white sorghum dough. Sequencing was performed using the Illumina MiSeq platform. The assembly yielded a gapped chromosome of 5,320,738 base pairs and an ungapped plasmid of 30,676 base pairs encoding 5,468 genes.

## ANNOUNCEMENT

*Bacillus cereus* is traditionally recognized as a pathogen associated with foodborne diseases; however, certain strains have been identified with potential probiotic properties, such as the production of bioactive compounds and antimicrobial substances (1, 2). This dual nature creates both opportunities and challenges in its application as a probiotic.

Sorghum is an important crop in Mexico, with the northeastern state of Tamaulipas being the largest producer. Although most production corresponds to red sorghum used as cattle feed, several white sorghum genotypes suitable for human consumption have recently been developed (3). The popularity of sorghum-based foods as a potential substitute for or complement to corn in traditional foods, such as tortillas, is growing in the country (4).

In 2022, white sorghum var. *costeño* was obtained from the INIFAP experimental field in Río Bravo, Tamaulipas. Grains were cleaned of debris, nixtamalized in a 0.33 M Ca(OH)₂ solution at 70°C, boiled for 10 min, and then incubated at room temperature for 12 h. The pericarp was removed by washing. The dough was prepared using nixtamalized grains in a mill. The fermented beverage was prepared with 30 g of dough in 200 mL of distilled water. The mixture was incubated at 37°C and 100 rpm for 48 h. Beverage samples were plated on MRS agar (Becton Dickinson, Franklin Lakes, NJ, USA) and incubated for 24 h at 37°C. Individual colonies were re-cultured using the same conditions described above in MRS broth for purification and stock maintenance. DNA was extracted from bacterial MRS broth cultures using the DNeasy Blood and Tissue kit (Qiagen), gram-positive protocol, and quality was assessed with an Agilent 2100 Bioanalyzer.

Genome sequencing was performed at the Laboratory of Genomic Services (LABSERGEN-CINVESTAV, Irapuato, Mexico). A paired-end library was prepared with the Nextera DNA Flex Library Prep Kit (Illumina, San Diego, CA, USA) and sequenced on the Illumina MiSeq platform to generate 300-bp paired-end reads. Sequencing produced 1,064,797 read pairs, totaling 286.9 Mbp.

Read quality was verified using FastQC v.0.12.0 (5). Reads passing quality control were assembled with SPAdes v.3.15.4 (6), yielding 54 contigs (N50: 188.3 kb). Scaffolding was performed with RagTag v.2.10 (7) using *B. cereus* strain FDAARGOS_1084 (GenBank no. CP068135.1) as a reference. A draft chromosome assembly of 5,320,738 bp (G+C: 35.04%) was obtained. A plasmid of 30,676 bp (G+C: 35.34%) was constructed with plasmidSPAdes v.4.2.0 (8). Annotation was performed using the NCBI prokaryotic genome annotation pipeline (v. 6.10) (9), revealing 5,468 genes on the chromosome. Taxonomic identification was carried out using the PubMLST database (10), analyzing housekeeping genes (*glp*, *gmk*, *ilv*, *pta*, *pur*, *pyc,* and *tpi*) according to the scheme developed by Tourasse et al. (11) for the *B. cereus sensu lato* group. Default parameters were used for assembly and annotation, unless otherwise specified.

## Data Availability

The raw sequencing data for *Bacillus cereus* strain *riobravensis* have been deposited in the NCBI under BioProject accession number PRJNA939842 and Sequence Read Archive accession number SRX28925042. The draft chromosome assembly was deposited in GenBank under accession no. CP186337.1 and the plasmid under accession no. CP186338.1.
